# Berberine Reshapes the Balance of the Local Renin-Angiotensin System by Modulating Autophagy under Metabolic Stress in Pancreatic Islets

**DOI:** 10.1155/2021/9928986

**Published:** 2021-08-03

**Authors:** Chenghu Huang, Pan Lei, Caibi Peng, Min Li, Yifei Guo, Xuefeng Li

**Affiliations:** ^1^Department of Endocrinology, Taihe Hospital, Hubei University of Medical, Shiyan, 442000 Hubei, China; ^2^Department of Endocrinology, Bishan Hospital of Chongqing, Bishan, Chongqing 402760, China

## Abstract

**Results:**

Prolonged exposure to palmitate increased the expression of ACE and AngII type 1 receptor (ATR1) and decreased the ACE2 expression, which was partly offset by berberine. In ob/ob mice, berberine increased in tolerance to glucose, improved abnormal *β*-cell and *α*-cell distributions, upregulated ACE2 expression, and decreased autophagosomes and the expression of LC3 and SQSTM1/p62. Autophagosomes and expression of LC3 and SQSTM1/p62 were increased in ACE2KO mice.

**Conclusions:**

We demonstrated that berberine may improve the pancreatic islet function by regulating local RAS-mediated autophagy under metabolic stress.

## 1. Introduction

Obesity, which is due to a chronic imbalance between energy “input” and “output”, has become a major public health problem because of its epidemic proportions worldwide [1]. Extensive research has proven that the renin-angiotensin system (RAS) is strongly associated with the energy imbalance and organ dysfunction caused by obesity [2, 3]. In addition to the systemic RAS that modulates body fluid and cardiovascular homeostasis, the concept of a tissue RAS, located within individual tissue types to regulate the local organ function, is now well recognized [3]. Complete tissue RAS has been identified in the endocrine and exocrine pancreas, and the expression of its various components has been demonstrated in the islets of Langerhans [3]. The RAS consists of dual distinct and counterregulating axes. Classically, the angiotensin-converting enzyme (ACE)/Ang II (angiotensin II)/AngII type 1 receptor (ATR1) axis is responsible for the potent vasoconstriction, proinflammatory, prooxidative, proliferative, and hypertrophic effects. The ACE2/Ang-(1–7)/Mas receptor (Mas) axis constitutes an alternative axis that represents an intrinsic mechanism for inducing inverse actions by regulating the ACE/Ang II/ATR1 axis, thus inducing many beneficial effects in energy balance and beta-cell protection [4]. Metabolic stress is an important trigger for the RAS imbalance in the activation of the ACE/Ang II/ATR1 axis and inhibition of the ACE2/Ang-(1–7)/Mas axis, which can be reversed by ATR1 antagonists or Ang (1–7) [5, 6]. Our previous study further confirmed that the ACE2/Ang-(1–7)/Mas axis is one of the intraislet paracrine mechanisms of communication between *α-* and *β*-cells that ameliorates *β*-cell dedifferentiation and dysfunction induced by metabolic stress [5]. Therefore, the RAS balance plays a crucial role in modulating metabolic processes and represents a promising target for the treatment of metabolic disease.

The autophagy-lysosome pathway is both induced by obesity and implicated in the development of type 2 diabetes [7, 8]. Autophagy is crucial for various biological events, such as cellular remodeling during development and differentiation, adaptation to stress conditions, and extension of lifespan [9]. Metabolic stress (mouse models of which include high-fat diet-fed mice and ob/ob mice) induces an increase in microtubule-associated protein 1, light chain 3 (LC3), which plays a central role in the autophagy pathway [10]. Selective autophagy substrate sequestosome 1 (SQSTM1, also known as p62) mediates the specific recognition of ubiquitinated protein aggregates by binding to LC3 and is then scavenged by autophagy. In addition, in animal models of an obese or a diabetic phenotype, global and pancreas-specific deletions and/or mutations of certain autophagy genes (such as Atg7, Lamp2, p62) result in elevated blood levels of glucose, glucose intolerance, smaller increases in *β*-cell mass, and degenerative changes in pancreatic islets [8, 11]. Importantly, it has become increasingly evident that autophagy may be considered the linchpin linking the biological effect of the RAS and the quintessential regulator of RAS balance. The disturbance of RAS balance (increased Ang II and activation of its downstream signaling) increases autophagosome formation via NOX and ROS production [12, 13]. And some evidence shows that the inhibition of ATR1 receptor ameliorates hyperlipidemia and liver steatosis in type 2 diabetic db/db mice via stimulating autophagy [14]. However, a substantial gap in the literature remains regarding how autophagy signaling influences the balance of RAS, especially in pancreatic islets.

Berberine is a natural compound isolated from plants such as *Coptis chinensis* and *Hydrastis canadensis* and has multiple pharmacological activities, such as antimicrobial, antidiabetic, antihyperlipidemic, anti-inflammatory, and antioxidant properties [15–17]. Importantly, berberine, used as a nonclassic AMP-activated protein kinase (AMPK) agonist to upregulate autophagy, treats diabetes mellitus via antioxidant and anti-inflammatory activities [18]. In this study, we hypothesized that berberine would reshape the local RAS balance to ameliorate the development of obesity and promote islet function via the modulation of autophagy, and we utilized berberine as a tool for mechanistic studies as well as a possible active ingredient against pancreatic islet dysfunction.

## 2. Materials and Methods

### 2.1. Animals

Six-week-old ob/ob male mice (C57BL/6J background) and wild-type C57BL/6J male control mice were maintained under standard light conditions (12/12 h light/dark cycle) and were allowed free access to food and water. Wild-type C57BL/6 J and ob/ob mice control mice were purchased from Beijing HFK Bio-Technology Co., Ltd. (Beijing, China). Male ACE2-knockout (ACE2KO) mice (on a C57BL/6 J background) were obtained from the Institute of Laboratory Animal Science, Chinese Academy of Medical Sciences. The mice were fed a standard rodent chow diet (low-fat diet; 20% protein, 70% carbohydrate and 10% fat) which purchased from Beijing HFK Bio-Technology Co., Ltd. (Beijing, China). The care and experimental treatment of the animals was approved by the Animal Research Committee of Hubei Medical College, Shiyan, China. ob/ob mice were randomized to either a vehicle-treated (0.5% carboxymethyl cellulose) (ob/ob) or berberine-treated (100 mg/kg/d berberine plus 0.5% carboxymethyl cellulose) group (Ob-BBR) (Sigma-Aldrich, MO, US). Body weight, dietary intake, and water intake were recorded twice a week. The total calorie intake was calculated according to dietary calorie intake and expressed as g/mice/d. After 8 weeks of administration of intragastric berberine, the animals were weighed and anesthetized by intraperitoneal injection of pentobarbital at a dose of 0.6 mg/kg. Beginning at 6 weeks of age, ACE2KO mice were fed a low-fat diet for 16 weeks according to previously described methods [5].

### 2.2. Metabolic Measurements

At sacrifice, ~1 ml of blood was collected from the orbital vein under anesthesia. Plasma insulin concentrations were determined using an insulin enzyme-linked immunosorbent assay (ELISA) kit (Millipore, Billerica, MA, USA). For intraperitoneal glucose tolerance tests (IPGTT), after 4 and 8 weeks on their respective treatment, mice were fasted overnight and injected intraperitoneally (i.p.) with 2 g/kg glucose the following morning. Blood glucose was measured with an UltraTouch glucometer from cut tail tips at 0, 15, 30, 60, 90, and 120 min following glucose injection.

### 2.3. Islet Isolation and Functional Studies

Islets in wild-type control C57BL/6 J mice were isolated from the pancreas according to previously described methods [19]. The pancreas was perfused through the common bile duct with 1.5 mg/mL collagenase P (Roche Applied Science, Mannheim, Germany). The islets were picked by hand, and procedures were performed according to the steps of the functional studies. Islets were placed in 24-well plates with 25 islets per well. Palmitate solution was prepared as described previously [20]. In addition, to test the effect of berberine, experiments were performed in the absence or presence of berberine (10 *μ*M) in accordance with previous literature [21].

### 2.4. Analyses of the mRNA Expression Using Real-Time PCR

The total RNA from isolated islets and mouse pancreatic tissues was extracted using TRIzol Reagent (Invitrogen, Carlsbad, CA, USA). Real-time quantitative polymerase chain reaction (QPCR) was performed using a LightCycler (Roche Diagnostics GmbH, Mannheim, Germany). The relative transcript levels were normalized to 36B4 and calculated using the 2^−∆∆CT^ statistical method. The PCR primer sequences used were as follows: ACE (forward primer: 5′-TGA GAA AAG CAC GGA GGT ATC C-3′ and reverse primer: 5′-AGA GTT TTG AAA GTT GCT CAC ATC A-3′), ATR1 (forward primer: 5′-CCA TTG TCC ACC CGA TGA AG-3′ and reverse primer: 5′-TGC AGG TGA CTT TGG CCA C-3′), ACE2 (forward primer: 5′-GCA CTC TCA GCA GAC AAG AAC AA-3′ and reverse primer: 5′-ATT TCA TCC AAT CCT GGC TCA AGT-3′), and Mas (forward primer: 5′-ATT TCA TCC AAT CCT GGC TCA AGT-3′ and reverse primer: 5′-GAC TAA CGA TGC CAC CGA TGC-3′).

### 2.5. Western Blotting Analysis

Protein from 300 islets was extracted using CytoBuster Protein Extraction Reagent (Beyotime Institute of Biotechnology, Beijing, China). Western blotting was performed as previously described [22]. Protein samples (30 *μ*g) were separated by 8% SDS-polyacrylamide gel electrophoresis and transferred onto pure nitrocellulose membranes (0.45 mm; Bio-Rad, Hercules, CA, USA). Membranes were blocked and incubated with one of the specific antibodies overnight at 4°C. Blots were probed with peroxidase-conjugated goat anti-mouse *β*-actin (Pierce Biotechnology, Rockford, USA) or peroxidase-conjugated goat anti-rabbit ACE2 (1 : 500; Abcam, Cambridge, MA, USA) and ACE (1 : 500; Abcam, Cambridge, MA, USA) for 1 h at room temperature followed by chemiluminescence detection (Amersham Pharmacia Biotech, Piscataway, NJ, USA).

### 2.6. Immunohistochemistry

Paraffin sections (5 *μ*m) were rehydrated, and antigen retrieval was performed using a PickCell pressure cooker. The primary antibodies used were guinea pig anti-insulin (1 : 150; Abcam, Cambridge, MA, USA), rabbit anti-ACE2 (1 : 200; Abcam, Cambridge, MA, USA), and rabbit anti-ACE (1 : 200; Abcam, Cambridge, MA, USA). Secondary antibodies were conjugated to Alexa Fluor 488 (Jackson ImmunoResearch Laboratories, West Grove, PA, US) or Dylight 549 (Abbkine, CA, US). The nuclear counterstain 4′6′-diamidino-2-phenylindole (DAPI, 1 : 1000; Invitrogen, Carlsbad, CA, USA) was also used. All of the digital images were acquired using a fluorescence microscope equipped with a DC 200 digital camera (C-1/TE200U, Nikon, Tokyo, Japan) and were subsequently analyzed using Image-Pro Plus software version 5.0 (Media Cybernetics). The density threshold selection tool was used to select the pancreatic islet areas marked with insulin and glucagon, which were depicted as a percentage of the mean islet cross-sectional area [23]. Additionally, pancreatic sections were stained for LC3 (1 : 500; Medical and Biological Laboratories Co, Ltd, Woburn, MA, USA) or p62/SQST1 (1 : 500; Abcam, Cambridge, MA, USA) using anti-rabbit primary antibodies. HRP-conjugated goat anti-rabbit IgG (1 : 200; Servicebio, Wuhan, Hubei, China) was used as a secondary antibody at a 1 : 100 dilution. All sections were analyzed using light microscopy, and images were captured using a computer image analysis system.

### 2.7. Transmission Electron Microscopy

Pancreatic sections were fixed with 4% glutaraldehyde and postfixed in 1% osmium tetroxide at 4°C. The samples were subsequently washed again, dehydrated with graded alcohol, and embedded in epon-araldite resin. Ultrathin 50 nm sections were obtained using an ultramicrotome. Sections were then stained with uranyl acetate and lead citrate. A Hitachi H-7500 transmission electron microscope (TEM) was used to observe autophagosomes.

### 2.8. Statistics

Results are expressed as the mean ± SEM. Statistical analysis was performed using the SPSS statistical analysis program. The statistical significance of quantitative results was evaluated using an analysis of variance (ANOVA) test. A 2-tailed *p* value of less than 0.05 was considered statistically significant.

## 3. Results

### 3.1. Palmitate Induced an Imbalance in the RAS in Pancreatic Islets In Vitro

**Figure 1:**
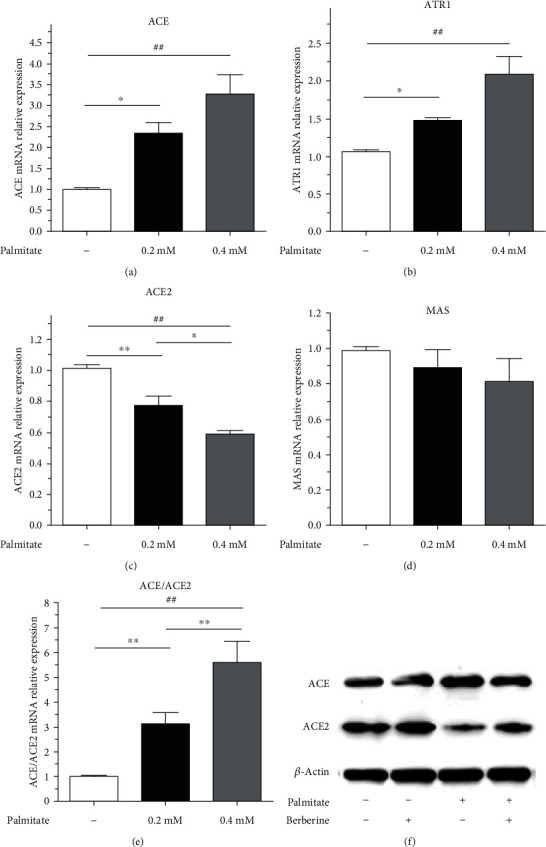
Palmitate triggered an imbalance in the RAS in a concentration-dependent manner. After islet cells were incubated with palmitate for the indicated durations, ACE (a), ATR1 (b), ACE2 (c), Mas (d), and ACE/ACE2 (e) mRNA expression was determined by QPCR. (f) The ACE and ACE2 protein expression was determined by immunoblotting. Data are shown as the mean ± SEM of three independent experiments. ^∗^*p* < 0:05; ^∗∗^*p* < 0:01;^∗∗∗^*p* < 0:001.

To further explore the effect of metabolic stress on the expression of the RAS, we detected the expression of RAS components in culture with palmitic acid. When islet cells were preincubated with palmitate at 0.2 or 0.4 mmol/L for a period of 48 h, The ACE mRNA expression was increased 2.32- or 3.27-fold, respectively (Figure 1(a)). The expression of ATR1 increased synchronously with ACE (*p* < 0.05); thus, palmitate induced the activation of the ACE/ATR1 axis (Figure 1(b)). However, with the administration of palmitate at 0.2 or 0.4 mmol/L, the ACE2 mRNA expression was markedly decreased by 23% or 41%, respectively (*p* < 0.05) (Figure 1(c)). Interestingly, the expression of Mas and ACE2 mRNA had the same trend but without statistical significance (*p* > 0.05) (Figure 1(d)), whereas with the expression of ACE and ACE2, the increase in ACE/ACE2 was readily apparent (*p* < 0.05) (Figure 1(e)). To further confirm the effect of palmitate on the RAS, we administered 0.4 mM palmitate to cultured islet cells in a subsequent study. Similar to the mRNA expression, the ACE expression was significantly upregulated, whereas the ACE2 expression was clearly decreased (Figure 1(f)). These results indicate that palmitate activates the ACE/ATR1 axis and inhibits the ACE2/Mas axis dependent on palmitate concentration in isolated islets.

### 3.2. Berberine Reshaped the Balance of Local RAS in Pancreatic Islets

**Figure 2:**
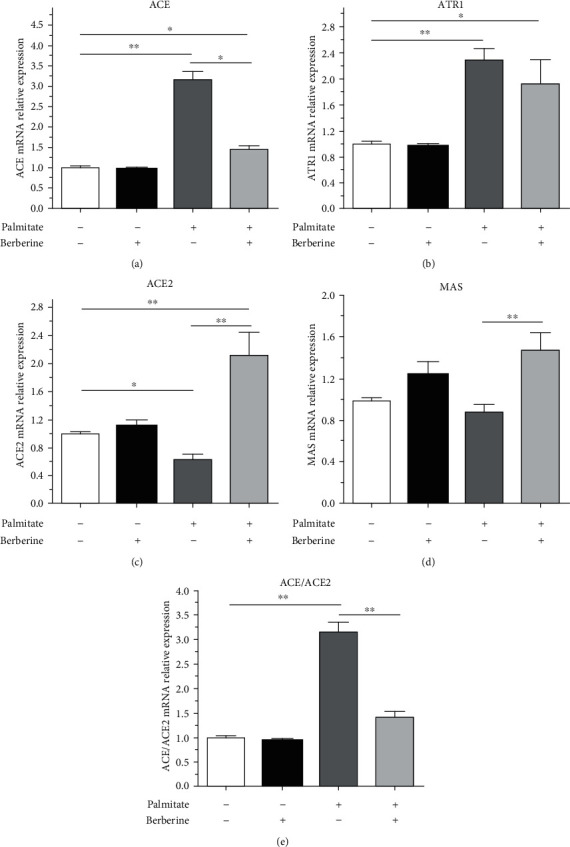
Berberine attenuated the disorder of the RAS. (a)–(e) After islet cells were incubated with berberine and palmitate, ACE (a), ATR1 (b), ACE2 (c), and Mas (d) mRNA expression was determined by QPCR. Data are shown as the mean ± SEM of three independent experiments. ^∗^*p* < 0.05; ^∗∗^*p* < 0.01.

We administered berberine 10 *μ*M to islet cells preincubated with palmitate to investigate the effect of berberine on RAS balance. Relative to islet cells preincubated with palmitate at 0.4 mmol/L for a period of 48 h, berberine could significantly decrease the ACE mRNA expression by 55.07% (*p* < 0.05) (Figure 2(a)). And berberine slightly inhibited the ATR1 mRNA expression by 15.84% (Figure 2(b)). However, berberine does not reduce the expression of ACE and ATR1 to normal levels, indicating that the malignant effects of palmitate could be only partially reversed. Importantly, with respect to palmitate alone, berberine greatly enhanced the ACE2 mRNA expression by 3.27-fold, even more than that of the normal control group (*p* < 0.05) (Figure 2(c)). And Berberine has little effect on the Mas mRNA expression (Figure 1(d)). Thus, the effects of berberine on the ACE/ACE2 ratio were more obvious (Figure 2(e)). Analogous to the mRNA expression results, berberine sharply inhibited the protein expression of ACE and dramatically increased that of ACE2 (Figure 1(f)).

### 3.3. Berberine Ameliorated Metabolic Parameters and Structural Disorders

**Figure 3:**
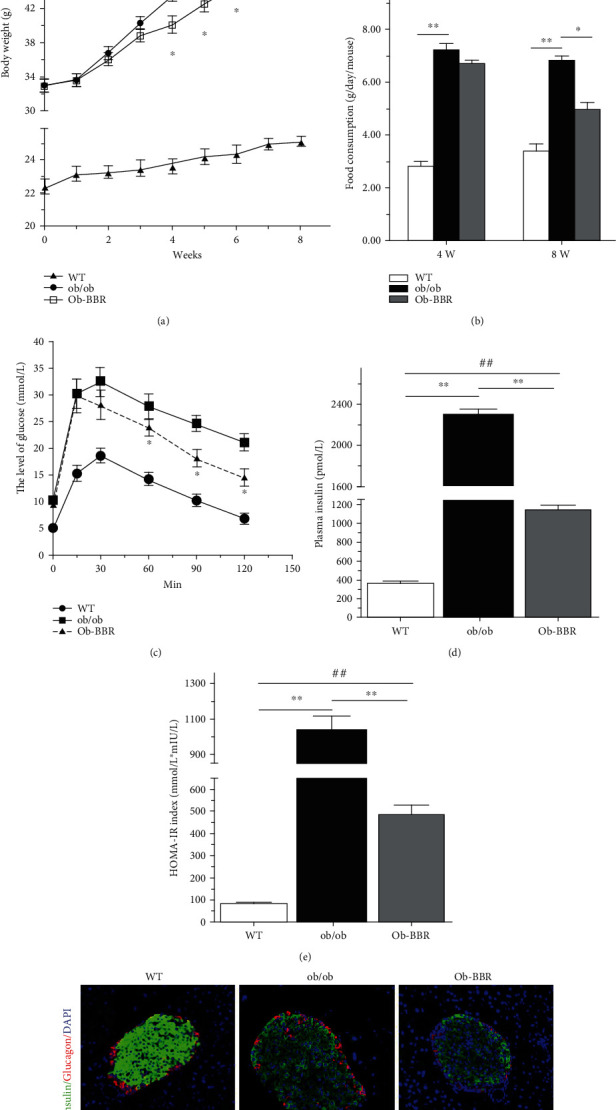
Berberine ameliorated metabolic parameters. (a) Effect of berberine on body weight. (b) Effect of berberine on food intake. (c) Intraperitoneal glucose and insulin tolerance tests (IPGTTs). Serum glucose levels were determined in overnight-fasted or 6-hour-fasted mice of each genotype (4 and 8 weeks). (d) Insulin levels from vehicle-treated mice and BBR-treated mice. Serum insulin levels were determined in overnight-fasted mice of each genotype by ELISA (8 weeks). (e) The homeostatic model assessment of insulin resistance (HOMA-IR) index. HOMA − IR = fasting glucose (mmol/L) × fasting insulin (mU/L)/22.5. (f) Representative images of immunofluorescence staining for insulin and glucagon (×400). WT (*n* = 6): wild-type C57BL/6 J mice; ob/ob (*n* = 6): ob/ob mice; Ob-BBR (*n* = 7): berberine-treated ob/ob mice. Data were expressed as the mean ± SEM, ^∗^*p* < 0.05, ^∗∗^*p* < 0.01 vs. ob/ob group; ^##^*p* < 0.01 vs. WT group.

As expected, compared with wild-type C57BL/6 J mice, body weight and food intake (g/kg/h) were significantly greater in the ob/ob and Ob-BBR groups (Figures 3(a) and 3(b)). After four weeks of treatment with berberine, an obvious decrease in body weight of the ob/ob mice was observed that lasted until the end of the intervention period (Figure 3(a)). The IPGTT showed that ob/ob mice had an impaired tolerance to glucose, whereas berberine seemed to markedly improve the tolerance (Figure 3(c)). ELISA showed that the plasma insulin concentration in the ob/ob group was significantly higher than that in the WT group, with a 6.13-fold increase, and was nearly twice that of the Ob-BBR group (Figure 3(d)). To further investigate the effect of berberine on insulin resistance, HOMA-IR (Homeostasis model for assessment of insulin resistance) was calculated, and we found that berberine partly decreased HOMA-IR in the Ob-BBR group (Figure 3(e)). Immunofluorescence showed that the islet structure of insulin and glucagon cells keeps the islets intact in ob/ob mice (Figure 3(f)). Unlike the defined *α*-cell mantle and *β*-cell core characteristic of islets in wild-type C57BL/6 J mice, islets of ob/ob mice appeared to maintain a more scattered organization and a higher percentage of *α*-cells. The increase in *α*-cell mass did not occur with berberine treatment, and in this group, the *α*/*β* ratio was evidently lower.

### 3.4. Autophagy Might Mediate the Protective Effect of Berberine

**Figure 4:**
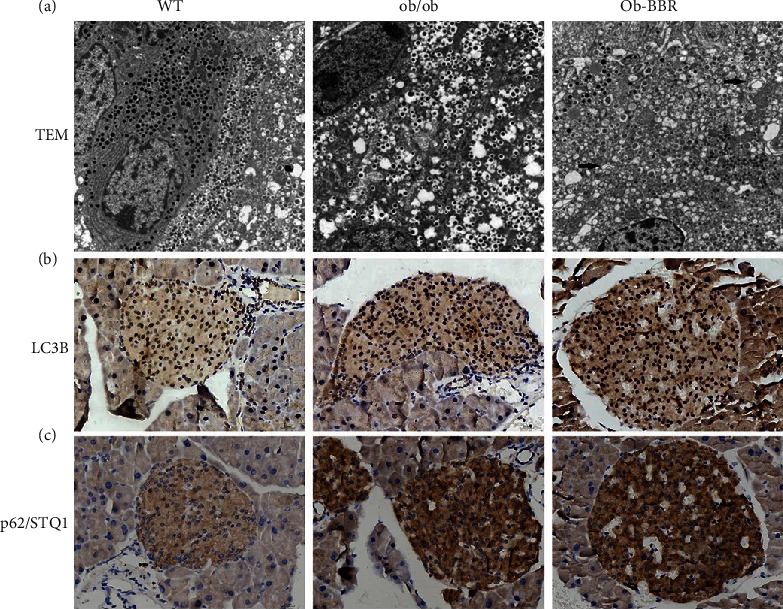
Autophagy mediated the protective effect of berberine in pancreatic islet cells. (a) Representative images of the islet cell structure by TEM. The black arrows show autophagosomes. (b, c) Representative images of the islet cell structure by immunohistochemistry (×400). WT (*n* = 7): wild-type C57BL/6 J mice; ACE2KO (*n* = 7): ACE2KO mice. The arrows show autophagy.

We further verified that autophagy was involved in the berberine-mediated protective effect on pancreatic islets by transmission electron microscope (TEM) (Figure 4). Ultrastructural changes in TEM images in the two ob/ob mouse groups displayed aberrant cytoplasm characterized by mitochondria, endoplasmic reticulum, free ribosomes, and irregular nuclei, as well as few autophagosomes and lysosomes. TEM analysis demonstrated a large number of electron-dense homogeneous spherical granules in the ob/ob groups, while the Ob-BBR group demonstrated a decreased proportion of paler and less dense granules (Figure 4(a)). A decreased proportion of autophagosomes and lysosomes in the pancreatic islet of the Ob-BBR group signified that berberine promoted autophagy. We also detected the intracellular localization and expression of LC3 and SQSTM1/p62 by immunohistochemistry (Figures 4(b) and 4(c)). Interestingly, the protein expression of LC3 showed the same trend as p62 in pancreatic islets. In addition, they were both increased in the ob/ob group and could be further induced by berberine in the Ob-BBR group. However, LC3 localized in the nucleus and p62 expression was concentrated in the cytoplasm.

### 3.5. Knockout of ACE2-Induced Autophagy Formation In Vivo

**Figure 5:**
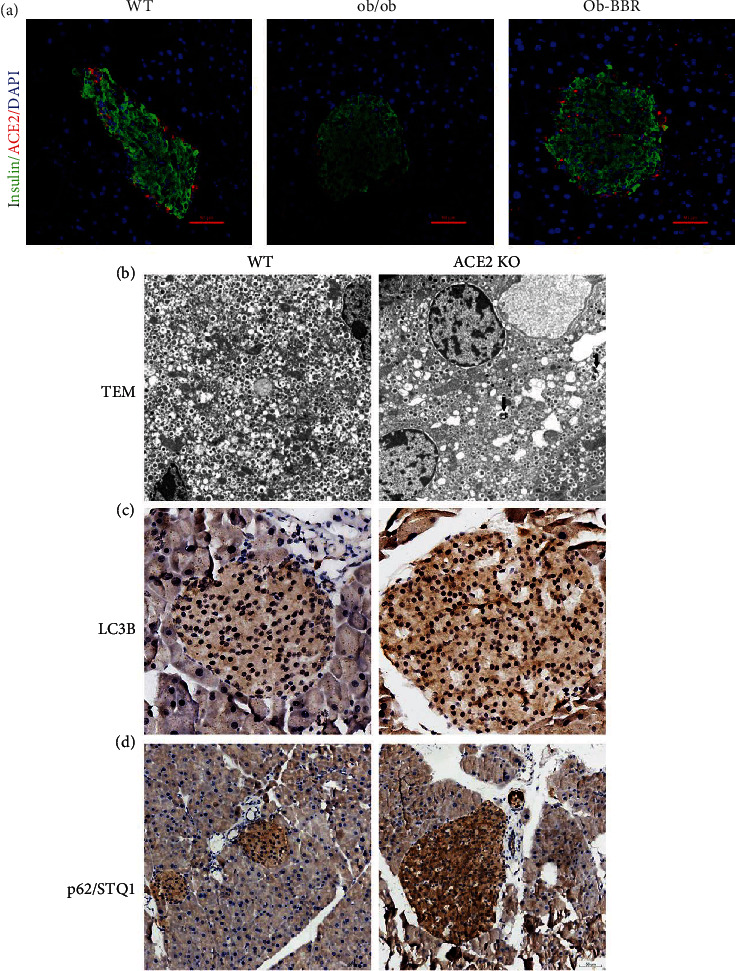
Knockout of ACE2-induced autophagy formation in vivo. (a) Representative images of immunofluorescence staining for insulin (green) and ACE2 (red) (×400). WT (*n* = 6): wild type C57BL/6 J mice; ob/ob (*n* = 6): ob/ob mice; Ob-BBR (*n* = 7): berberine-treated ob/ob mice. (b) Representative images of the islet cell structure by TEM. The arrows show autophagosomes. (c, d) Representative images of the islet cell structure by immunohistochemistry (×400). WT (*n* = 7): wild type C57BL/6 J mice; ACE2 KO (*n* = 7): ACE2 KO mice.

To confirm whether the RAS is involved in the effect of berberine on autophagy in pancreatic islets, we double immunostained pancreatic sections from the two ob/ob mouse groups. Interestingly, the ACE2-positive cells were mostly localized towards the islet periphery, which was the position of *α*-cells as shown in our previous study [5]. In addition, ACE2-positive cells were increased in the Ob-BBR group (Figure 5(a)). These results demonstrated that berberine ameliorates the metabolic stress-induced imbalance of the local RAS by the decrease in ACE2. Thus, we further explored whether autophagy was involved in the berberine-mediated protective effect on pancreatic islets in ACE2 knockout (ACE2 KO) mice. Ultrastructural changes in TEM images in ACE2 KO mice displayed a few autophagosomes and lysosomes (Figure 5(b)). We also found that the expression of LC3 and SQSTM1/p62 was increased in ACE2 KO mice (Figure 5(c)).

## 4. Discussion

Autophagy is likewise a physiological survival response that removes aberrantly long-lived proteins and damaged organelles in order to maintain cytoplasmic quality and promote cell survival under stress/starvation.

Autophagy acts as a central regulator of berberine-mediated effects [24–26]. It is triggered by stress through different mechanisms, which induces autophagosomes to fuse with lysosomes to form autolysosomes, resulting in degradation of the cargo. Autophagy is therefore a very important process for survival. The protein microtubule-associated protein 1, light chain 3 (LC3), functions in autophagosome formation and plays a central role in the autophagy pathway [27]. Under basal conditions, LC3 is known to exist in a soluble form termed LC3-I, and upon upregulation of the autophagy pathway, LC3 is converted to a lipidated form termed LC3-II that associates with autophagosomal membranes. In our study, in ob/ob mice, berberine increased in tolerance to glucose, improved abnormal *β*-cell and *α*-cell distributions, upregulated ACE2 expression, and decreased autophagosomes and lysosomes and the expression of LC3 and SQSTM1/p62. And the activation of autophagy was observed in pancreatic islets of ACE2-deficient mice, as indicated by increased numbers of autophagolysosomes and the expression of LC3 and SQSTM1/p62. We noticed that AMPK-dependent autophagy is the main target of the berberine treatment effects [28, 29]. AMPK is an important downstream regulator of ACE2-mediated protective effects [30]. Recent studies have demonstrated that the inhibition of ATR1 relies on the AMPK pathway to activate autophagy [31].

Autophagy was originally defined as the process of degradation and recycling of proteins, other macromolecules, and organelles. Autophagy deficiency has been reported to cause structural abnormalities and *β*-cell dysfunction [8, 32]. The major findings of the present study are that metabolic stress induces an imbalance in the local RAS in pancreatic islets, including an increase in the ACE/ATR1 axis and inhibition of the ACE2/Mas axis, which can be reversed by berberine. ACE2 knockout facilitates the formation of autophagosomes and the expression of autophagy-associated proteins LC3 and p62. Furthermore, berberine decreases metabolic stress-induced autophagy to ameliorate metabolic parameters and structural disorders. Here, we rationalize the idea that autophagy may mediate how berberine reshapes the balance of the local RAS in pancreatic islets.

Berberine has been found to effectively and safely improve *β*-cell function and attenuate insulin resistance in high-fat diet-fed and diabetic mice [33, 34]. In the present metabolic stress model, we used TEM and H&E staining and found that long-term treatment with berberine induced a significant increase in the tolerance to glucose and a decrease in body weight and lipid ectopic accumulation in ob/ob mice. These data suggested that berberine effectively abolishes pancreatic islet dysfunction during metabolic stress. Interestingly, after 8 weeks of treatment in ob/ob mice, it seems paradoxical that berberine decreased the plasma insulin levels detected by ELISA while increasing the insulin protein expression in *β*-cells as shown by immunofluorescence. However, these results are very reasonable because metabolic stress, such as obesity, can lead to hyperinsulinemia also induce *β*-cell dedifferentiation and apoptosis [35–37]. Collectively, these findings are consistent with previous studies showing that berberine has the potential to act as a therapeutic agent for obesity and type 2 diabetes [38].

The RAS is a pluripotent toxic factor and key contributor in insulin resistance and subsequent *β*-cell dysfunction [39]. Under metabolic pressure, ATR1 blockade or activation of ACE2/Ang [1–7] not only attenuates pancreatic *β*-cell dedifferentiation and apoptosis but also promotes islet remodeling and glucose homeostasis [5, 40]. To our knowledge, this study demonstrated that berberine reshapes the balance of the local RAS in pancreatic islets, including inhibition of the ACE/ATR1 axis and activation of the ACE2/Mas axis. Reviewing the current literature, the possible mechanisms of the berberine-induced protective effects lie in the inhibition of the ACE/ATR1 axis. Berberine regulates Ang II-induced proliferation, collagen synthesis, and cytokine secretion of cardiac fibroblasts via the AMPK-mTOR-p70S6K signaling pathway [41]. Ko found that berberine abolished the generation of reactive oxygen species and MCP-1 expression induced by Ang II in human umbilical vein endothelial cells in a dose-dependent manner [42]. Further, berberine was found to be a direct dual inhibitor of *α*-glucosidase and ACE [43]. Furthermore, our data first found that berberine increased the ACE2 expression, which may be related to the suppression of the ACE/ATR1 axis [44]. Together, these data confirm the causal role of berberine-remodeling in the local RAS in the pancreatic islet.

The limitations of this study should be noted. Although we have shown that autophagy is a target of berberine, the effects of the local RAS on autophagy in ACE2 KO high-fat diet-fed diabetic mice were not investigated. Additionally, the AMPK pathway, a classic downstream target of berberine and the RAS, was not used in this study. Further studies are needed to verify the above mechanisms.

In conclusion, obesity increased ACE1/ATR1 activity and decreased the ACE2 expression, inducing RAS imbalance and *β*-cell function. Berberine inhibited the ACE1/ATR1 expression and autophagy, activated ACE2, and improved *β*-cell dysfunction. ACE2 KO mimicked RAS imbalance, which dysregulated the levels of LC3 and LAMP2 and increased the number of autophagolysosomes in high-fat diet-fed mice. Many questions regarding the molecular network underlying these responses must be answered. However, the possibility that RAS-mediated autophagy could play an essential role in berberine-induced improvements in *β*-cell dysfunction in metabolic stress is very exciting.

## Data Availability

No data were used to support this study.
